# Isolation and Genetic Identification of Endophytic Lactic Acid Bacteria From the Amazonian Açai Fruits: Probiotics Features of Selected Strains and Their Potential to Inhibit Pathogens

**DOI:** 10.3389/fmicb.2020.610524

**Published:** 2021-01-08

**Authors:** Suenne Taynah Abe Sato, Joana Montezano Marques, André da Luz de Freitas, Raphaela Cristina Sanches Progênio, Márcio Roberto Teixeira Nunes, Janaína Mota de Vasconcelos Massafra, Fábio Gomes Moura, Hervé Rogez

**Affiliations:** ^1^Centre for Valorization of Amazonian Bioactive Compounds (CVACBA), Universidade Federal do Pará, Belém, Brazil; ^2^Laboratório de Genômica e Bioinformática, Centro de Genômica e Biologia de Sistemas, Universidade Federal do Pará, Belém, Brazil; ^3^Centro de Inovações Tecnológicas (CIT), Centro Nacional de Primatas, Instituto Evandro Chagas (IEC), Belém, Brazil; ^4^Instituto de Ciências Biológicas (ICB), Biologia de Agentes Infecciosos e Parasitários (BAIP), Universidade Federal do Pará, Belém, Brazil

**Keywords:** *Euterpe oleracea*, *Lactiplantibacillus plantarum*, *Pediococcus pentosaceus*, probiotics, endophytic bacteria

## Abstract

The açai palm (*Euterpe oleracea*) is native to the Amazon basin, a humid tropical forest. High levels of total mesophilic bacteria with high diversity have been consistently reported in açai fruits. As local consumers have few digestive problems, the results of the present study reveal the lactic acid bacteria (LAB) recovered from açai fruits with characteristics that suggest they are possible candidates for probiotics and antagonistic potential against pathogens for the first time. Açai fruits were sampled from five different locations in the Eastern Amazonia floodplains. Sixty-six isolates were recovered from fruits and tested for some probiotic characteristics following FAO/WHO guidelines. Approximately 65% of the isolates showed no catalase or oxidase activity, Gram-positive staining or cocci and bacilli cell morphology. Furthermore, 48% of the isolates demonstrated preliminary characteristics that suggest safety for use, as they presented no coagulase enzyme activity or gamma-hemolysis. These strains were identified as belonging to the genera *Lactiplantibacillus* and *Pediococcus*, and 32 strains also presented resistance to vancomycin, ciprofloxacin and streptomycin. In addition, 28 isolates showed a survival rate, expressed as log cycle reduction, higher than 0.9 under gastric conditions (pH 2). All strains tested positive in bile salts deconjugation tests and showed a survival rate higher than 0.8 in the presence of this salt. Regarding antimicrobial activity against pathogens, all strains were able to inhibit *Salmonella* Typhimurium (ATCC^®^ 14028^TM^) and 97% were capable of inhibiting *Escherichia coli* (ATCC^®^ 25922^TM^). Concerning the results of *in vitro* antagonistic assays, three isolates (B125, B135, and Z183 strains) were selected for antagonistic tests using açai juice contaminated with these two pathogens. All tested LAB strains were able to inhibit pathogen growth in açai juice. In summary, açai fruits are a potential source of LAB isolates to be investigated as probiotics.

## Introduction

The Amazon has the ideal climatic conditions for plant growth, especially for many types of palm trees. Some of these palms are important for both the agricultural industry, which can offer a source of income for the local population, and for biotechnological purposes, which remains understudied. *Euterpe oleracea* Martius palm is native to the Amazon and is widely spread in the northern region of Brazil. The softened fruit of this plant is used to prepare a juice called açai, a typical food of the local population and has been well studied due to its high nutritional value and, importantly, its high levels of antioxidants (i.e., phenolic and tocopherol compounds) (Bichara and Rogez, 2011; Darnet et al., 2011).

The forms of consumption of açai in the Amazon estuary have been reported for several centuries (Wallace, 1853; Cavalcante, 1976) and have traditionally been seen until today. Most commonly, açai is consumed along with other foods that can be flour derived from cassava, fried fish, salted shrimp, among other protein foods. However, a large part of the population also consumes the naturally fermented acai drink (called “past açai” or “açai sour”), which can be consumed directly or used in the production of porridges. Interestingly, those who consume past açai do not show clinical signs of food intoxication, so this phenomenon may be due to spontaneous fermentation that is primarily performed by lactic acid bacteria (LAB), which are desirable for the safe production of different foods (Aguiar et al., 2013).

In the last decade, a variety of studies have focused on the açai microbiota. Rogez et al. (2012) demonstrated the presence of high levels of microorganisms immediately after the harvest of açai fruits with respect to molds and yeasts (1.11 × 10^5^ CFU g^–1^ DM) and total mesophilic bacteria (2.64 × 10^6^ CFU g^–1^ DM). Moura et al. (2018) investigated the diversity of the native bacterial community in açai fruits post-harvest and identified the most representative phyla as *Proteobacteria*, *Firmicutes*, *Actinobacteria*, *Bacteroidetes*, and *Acidobacteria*, with approximately 200 bacterial genera identified. However, in this study, to identify the diversity of bacteria, the authors used culture-independent methods based on the 16S rRNA gene. McCulloch et al. (2014) isolated and sequenced the LAB strain *Lactococcus lactis* AI06 from the mesocarp of açai fruits and suggested the possibility of it being a probiotic based on FAO/WHO (2006) criteria.

It is interesting to elucidate the microbiota of açai fruits, especially the endophytic LAB involved in its fermentation since many studies have shown that many fermented foods are rich sources of microorganisms with probiotic characteristics (Argyri et al., 2013; Barbosa et al., 2014; García-Ruiz et al., 2014; Oh and Jung, 2015; Domingos-Lopes et al., 2017). LAB comprise a major group of probiotics, and many LAB have received generally recognized as safe (GRAS) status from the American Food and Drug Administration (FDA), which involves not only the assessment of strain identity, safety, cell surface properties, their good survival during processing and shelf-life, but have to provide demonstration of a health beneficial effect when administered in proper quantities (Joint FAO/WHO, 2002; Hill et al., 2014; Kumar et al., 2015; Reid et al., 2019).

Several studies aiming to characterize microorganisms as probiotics and demonstrate their beneficial effects have been reported in the literature. To determine the probiotic properties of strains, it is recommended to perform preliminary *in vitro* tests that include tolerance tests under gut conditions until the colon (e.g., assessments of their viable physiological state and ability to adhere to the surface of the intestine cell) (FAO/WHO, 2001; Morelli, 2007), in addition to conferring specific health benefits, which is considered the final criterion for selecting a successful probiotic strain (Tuomola et al., 2001; Greppi et al., 2017). Common benefits include immune system regulation, the production of compounds with antioxidant activity, and competitive adherence capacity to the intestinal mucosa, which contributes to strengthening the gastrointestinal epithelium barrier and maintaining the positive balance of the beneficial microbiota in the gut via the production of antimicrobial compounds (primarily antimicrobial peptides and organic acids) and preventing the growth of pathogenic bacteria (Parvez et al., 2006; Jankovic et al., 2010; Li et al., 2014; Oh and Jung, 2015). LAB (e.g., *Lactiplantibacillus* and *Bifidobacterium* strains) have been reported to inhibit the growth of common pathogens responsible for food poisoning (Presti et al., 2015).

In the present study, endophytic LAB isolated from Amazonian açai fruits were identified and evaluated for the presence of some common characteristics of probiotics and antagonistic activity against pathogens. The probiotic characteristics evaluated in this study for the newly isolated LAB proceeded through *in vitro* experimentation. Subsequently, the isolates that passed the safety tests and probiotic characteristics were identified by 16S rRNA gene sequence analysis and based on the results of *in vitro* antimicrobial tests, three strains were investigated for antagonistic activity against foodborne enteric pathogens in açai juice.

## Materials and Methods

### Isolation and Pre-selection of LAB

**FIGURE 1:**
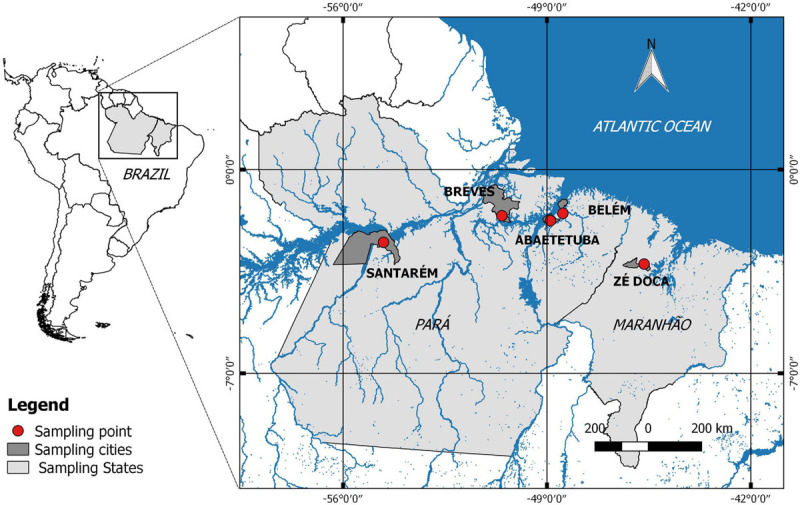
Map with marked açai fruit collection regions.

Endophytic strains were isolated from açai fruits collected in five locations of the Amazon estuary (Combu Island, Belém-PA, Abaetetuba-PA, Breves-PA, Santarém-PA, and Zé Doca-MA; Figure 1). Açai fruits (250 g) were superficially sanitized in 250 mL of a 70% (v/v) ethanolic solution for 1 min followed by a treatment in a 2.5% (v/v) sodium hypochlorite solution (NaClO) for 5 min, and a 70% (v/v) ethanolic solution for 1 min, after which the fruits were washed three times with sterile distilled water for 30 s each. The açai fruits were manually pulped by maceration in sterile stainless-steel sieves (2.5 mm mesh), after which 25 g of pulp was diluted in 225 mL of peptone water (H_2_Op) in a sterile bag. The samples were then serially diluted, and 1 mL of each sample (diluted from 10^–1^ to 10^–7^) was added to plates containing Man, Rogosa and Sharpe (MRS) agar. The plates were incubated for 48 h at 37°C under anaerobiosis. All isolates were subsequently purified using the depletion technique and preliminarily identified based on their morphological characteristics and Gram stain (gram-positive cocci or bacilli), negative catalase reaction (3% v/v H_2_O_2_) and cytochrome oxidase activity. The isolated LAB strains were designated by the first letter of the collection site for the açai plants (A—Abaetetuba, B—Breves, C—Combú Island—Belém, and Z—Zé Doca) followed by progressive isolation numbers.

### Safety Evaluation of LAB Isolates

For the coagulase test, 0.3 mL of culture containing each isolate was transferred to sterile tubes containing 0.3 mL of rabbit plasma (Coagu-Plasma LaborClin^®^, Pinhais/PR, Brazil) and incubated at 36 ± 1°C for 6 h. A result was considered positive when the formation of a large and organized clot or total clotting was observed (Perin et al., 2014).

Hemolytic activity was determined by inoculating the strains on Columbia agar plates containing 5% (w/v) of sheep blood, and after 48 h of incubation at 37°C, the strains were assessed according to the methodology proposed by Angmo et al. (2016). After colony growth, the plates were examined for signs of α-hemolysis (green zones around colonies), β-hemolysis (bright zones around colonies) or γ-hemolysis (no zone around colonies).

Antibiotic resistance was determined using the Kirby-Bauer method. Antibiotic discs (LaborClin^®^, Pinhais/PR, Brazil) containing cephalexin (30 μg), rifampicin (5 μg), chloramphenicol (30 μg), erythromycin (15 μg), vancomycin (30 μg), penicillin G (10 U), gentamicin (10 μg), tetracycline (30 μg), ampicillin (10 μg), ciprofloxacin (5 μg), streptomycin (10 μg), and clindamycin (2 μg) were used according to the recommendations of the Clinical and Laboratory Standards Institute (CLSI, 2012). The isolated LAB were grown on MRS agar overnight (18 h) at 37°C, and the strains were inoculated in 4 mL of sterile distilled water to achieve a standard turbidity No. 0.5 McFarland (Laborclin, Pinhais/PR, Brazil). Then, a swab was used to spread the inoculum over the surface of Muller Hinton agar, and the antibiotic discs were then applied to the plate. Antimicrobial susceptibility was assessed by measuring the zone of inhibition of bacterial growth after incubation at 37°C for 24 h. The assayed strains were compared to a standard provided by the manufacturer and classified as sensitive, intermediate and resistant. For erythromycin, the results were based on: Resistant ≤ 13 mm; Intermediate: 13–23 mm; Sensitive ≥ 23 mm. For gentamicin: Resistant ≤ 6 mm; Intermediate: 7–9 mm; Sensitive ≥ 10 mm. For vancomycin: Resistant ≤ 12 mm; Intermediate: 12–13 mm; Sensitive ≥ 13 mm. For other antibiotics: Sensitive ≥ 17.5; Intermediate: 12.5–17.4 mm; Resistant: ≤ 12.4 mm. The tests were also performed with a reference probiotic strain of *Lacticaseibacillus rhamnosus* (LMG 18243—Gent University, Belgium) as a positive control.

### Low pH Resistance

The resistance of strains to low pH conditions was tested as described by Oh and Jung (2015). Briefly, the isolated LAB were grown overnight, collected, adjusted to an optical density (OD_60__0 n__m_) of 0.5 (corresponding to 10^8^–10^9^ CFU/mL), resuspended in MRS broth adjusted to pH 2.0 (with a 4 M HCl solution), and then incubated at 37°C. Resistance was assessed by counting bacterial colonies on an MRS agar plate after 0 and 3 h of contact, reflecting the normal time spent by food in the stomach. MRS broth at pH 6.5 and the probiotic *L. rhamnosus* (LMG 18243) were used as controls. Isolates with survival rates, expressed as log cycle reduction, from 0.9 at pH 2.0 (Liu et al., 2013; Almeida Júnior et al., 2015; Ferrari et al., 2016) were selected for further testing. The rates were calculated according to the following equation (Eq. 1):

(1) $$S\,u\,r\,v\,i\,v\,a\,l\,r\,a\,t\,e=\frac{\log\mathrm{CFU}\,\mathrm{N1}}{\log\mathrm{CFU}\,\mathrm{N0}}$$

where N1 represents the total viable strain count after 3 h at pH 2.0, and N0 represents the total viable strain count at time 0 h.

### Resistance to Bile Salts

The resistance of strains to bile salt was tested as described by Tulini et al. (2013). Briefly, the isolated LAB were adjusted to OD_600 nm_ of 0.5 and inoculated in MRS broth containing 0.3% bile salts (Oxgall, Becton, Dickinson and Company, Franklin Lakes, United States). Cell viability was assessed by counting on an MRS agar plate, at 0 and 4 h (which reflects the time food requires to reach the small intestine) after incubating for 37°C. MRS broth samples without bile salts and *L. rhamnosus* (LMG 18243) were used as controls. Survival rates were also calculated as previously shown (Eq. 1). The isolates with a higher survival rate to 0.5 (Mathara et al., 2008) were selected.

### Ability to Deconjugate Bile Salts

The ability to deconjugate bile salts was tested according to a method adapted from Bautista-Gallego et al. (2013) by incubating each bacterial culture overnight on MRS agar plates containing 0.5% bile salts. After cultivation at 37°C for 48 h, bile salt hydrolase (BSH) activity was qualitatively evaluated by observing halos formed around the colonies or changes in colony morphology, according to Argyri et al. (2013), Solieri et al. (2014), and Ferrari et al. (2016). When halo formation was observed, their diameters were measured. As a control, plates were used without bile salts and tested with *L. rhamnosus* (LMG 18243).

### Antimicrobial Activity Against Pathogens

All strains were tested for antimicrobial activity against the pathogens *Escherichia coli* (LMG 17767, Gent University, Belgium), *Salmonella* Typhimurium ATCC^®^ 14028^TM^, *Enterococcus faecalis* ATCC^®^ 19433^TM^ and *Staphylococcus aureus* ATCC^®^ 25923^TM^ using the spot-on-the-lawn technique adapted from Barbosa et al. (2005). Briefly, 5 μL of each strain grown overnight in MRS broth was inoculated on MRS agar plates and incubated at 37°C for 24 h. After exposing the developed colonies to chloroform vapor for 30 min under UV light to induce cell death, the plates were aerated for 20 min and then overlaid with semisolid Brain Heart Infusion (BHI) culture medium (0.7% of bacteriological agar) inoculated with 100 μL (0.5%; approximately 10^8^–10^9^ CFU/mL) of the pathogenic indicator culture. Uninoculated MRS agar plates were used as a negative control, and the probiotic strain *L. rhamnosus* (LMG 18243) was used as the positive control. The zones of inhibition were measured in millimeters from the edge of the colony and expressed as the means. The correspondence between the symbols and the diameters were: (−) without inhibition, (+) zone of inhibition between 2 and 6 mm, (++) zone of inhibition between 7 and 11 mm and (+++) zone of inhibition between 12 and 16 mm.

### Identification of LAB Isolated From Açai Fruits

Twenty-eight of the 66 isolates were sequenced and identified in the present study at the genus and species level via 16S rRNA gene sequence analysis. Genomic DNA was extracted from each strain as described by Seldin and Dubnau (1985). The primers 8F (5′-AGA GTT GTA TCA TGG CTC AG-3′) and 1492R (5′-CGGTTA CCTTGT TAC GACTT-3′) were used to amplify the 16S rRNA gene. 2.5 mM MgCl_2_, 0.5 mM of each dNTPs, 1 U of Taq DNA polymerase, 1 × GoTaq^®^ Flexi Buffer (Promega, Madison, United States) and 0.2 mM of each primer. PCR amplification was performed in a final volume of 25 μL containing 1 × Green GoTaq^®^ Flexi Buffer (Promega^®^ ; Madison, United States), 2.5 mM MgCl_2_, 0.5 mM dNTPs, 0.2 μM each primer, 1 U of GoTaq^®^ (Promega), and 1 μL of template DNA (50–100 ng). PCR products were purified with ExoSAP-IT^®^ Express PCR Product Cleanup (Affymetrix, Santa Clara, CA) and sequenced using an Applied Biosystems 3500 Serial Genetic Analyzer (Applied Biosystems^®^) at the Evandro Chagas Technological Innovation Center (Belém, Brazil). The sequences were compared to those in the GenBank database using the BLAST algorithm (National Center for Biotechnology Information, Maryland, United States).

### Antagonistic Activity Against Pathogens in Açai Juice

The isolated strains showing the best results for the previous tests were assessed for their ability to inhibit the growth of pathogenic bacteria [*Escherichia coli* ATCC^®^ 25922^TM^ (EC) and *Salmonella* Typhimurium ATCC^®^ 14028^TM^ (ST)] in açai juice. For the experiments, the LAB grew overnight (in MRS broth, at 37°C, under anaerobiosis) and were adjusted to a final density (OD_600 nm_) of approximately 0.5 and resuspended in pasteurized açai at 82.5°C for 1 min. The pathogens were grown overnight in BHI broth (at 37°C, under anaerobiosis) and were also adjusted to a final density (OD_600 nm_) of approximately 0.5. The tests consisted of the following combinations: Açai + LAB and Açai + Pathogen in order to verify the viability of the only LAB, and only the pathogen in açai juice; and Açai + LAB + Pathogen for the antagonism test. The experiments were performed at room temperature (25°C). Cell viability was assessed by counting colonies on an MRS, Violet Red Bile (VRB), and Xylose Lysine Deoxycholate (XLD) agar plates for LAB, *E. coli* and *Salmonella* Typhimurium, respectively. The colonies were counted at the initial time (0 h), and after 24, 48, and 72 h. The plates were incubated under anaerobiosis at 37°C for 48 h.

### Investigation of LAB Tolerance at Different pHs

Each strain was cultured in MRS broth adjusted to pH 2.0, 2.5, 3.5, 4.5, 5.5, 6.5, 7.5, and 8.5. The tests were conducted in sterile 96-well flat-bottom microtiter plates, where each well was filled with 180 μL of the tested MRS broth and 20 μL of the culture with the OD_600 nm_ adjusted to 0.5. The optical density was recorded every 1 h for 12 h on a spectrophotometer at 600 nm. As a control, in addition to MRS broth at pH 6.5 (MRS broth without pH correction), the probiotic *L. rhamnosus* LMG 18243 was assessed. The plates in these assays were incubated at 37°C.

### Statistical Analysis

For the tests of resistance to low pH, bile salts and antagonistic activity against pathogens in acai juice, the data were analyzed by ANOVA (*p* < 0.05) and when significant, the means were compared by the Tukey test (*p* < 0.05). The data from the strain tolerance test at different pHs were analyzed by ANOVA (*p* < 0.05) and when significant, the average values in each time (from 1 to 12 h) were compared to the control time (0 h) by the Dunnet (*p* < 0.05).

## Results

### Isolation and Pre-selection of LAB

Sixty-six endophytic strains were isolated from açai fruit, 43 of which were selected for subsequent study taking into account their Gram positive, catalase negative and oxidase negative characteristics, among them 7 from Combú Island, Belém—PA (C21, C34, C35, C37, C38, C39, and C52), 6 from Abaetetuba—PA (A71, A72, A73, A74, A100, and A101), 24 from Breves—PA (B104, B105, B108, B109, B112, B113, B117, B118, B119, B120, B121, B122, B123, B125, B134, B135, B137, B138, B139, B140, B141, B142, B143, and B144), 2 from Santarém—PA (S150 and S153) and 4 from Zé Doca—MA (Z170, Z183, Z188, and Z190).

### Characterization of Safety Evaluation of LAB Isolates

In the hemolytic test, strains C34, C35, A100, A101, and B108 showed partial hemolysis of red blood cells and were classified as α-hemolytic, while strains A72, B104, B105, B117, B120, and S153 showed complete lysis of red cells (β-hemolytic). Only strains with non-hemolytic characteristics (32 strains) were subjected to the coagulase test, and they did not show coagulase activity in phenotypic tests.

**FIGURE 2:**
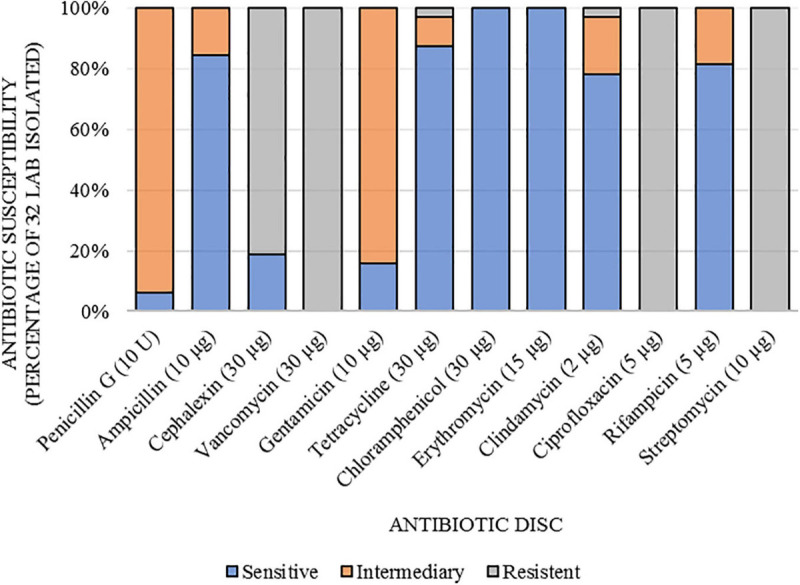
Susceptibility to antibiotics of lactic acid bacteria isolated from açai fruits (*Euterpe oleracea*).

The determination of antibiotic susceptibilities of *Lactobacillaceae* against 12 antibiotics is shown in Figure 2. The 32 strains were resistant to vancomycin (VAN 30 μg; 100%), ciprofloxacin (CIP 5 μg; 100%), and streptomycin (EST 10 μg; 100%). Most LAB were resistant to cephalexin (CEX 30 μg; 81%), except for B141, B142, B143, B144, S150, and Z183, which were susceptible. All strains were sensitive to chloramphenicol (CLO 30 μg; 100%), and erythromycin (ERI 15 μg; 100%). Regarding penicillin G (PEN 10 U), ampicillin (AMP 10 μg), gentamicin (GEN 10 μg), tetracycline (TET 30 μg), clindamycin (CLI 2 μg), and rifampicin (RIF 5 μg), the strains showed intermediate sensitivity or sensitivity, except for B137, which was resistant to tetracycline.

### Resistance to Low pH

The resistance of the strains to low pH conditions was evaluated *in vitro*. The viability of most assayed strains (97%) largely unchanged after 3 h at pH 2.0 (Supplementary Figure S1). Among the 32 strains evaluated, the viability of 11 (C39, B109, B122, B123, B125, B134, B135, B139, B142, Z170, and Z183) remained stable (*p* > 0.05) when compared to the initial number of viable cells (control), 9 strains (C21, C38, C52, A71, A73, A74, B118, B140, and B144) exhibited an increase in the number of viable cells (*p* < 0.05) and 12 strains exhibited reduced viability (*p* < 0.05) (C37, B112, B113, B119, B121, B137, B138, B141, B143, S150, Z188, and Z190).

Considering the survival rates of the strains evaluated at pH 2.0 (Table 1), of the 12 strains that showed a reduction (*p* < 0.05) in the number of viable cells, 8 still had a survival rate, expressed as log cycle reduction, above 0.90 (C37, B113, B137, B138, B141, B143, Z188, and Z190), 4 strains had viability below this rate [B112 (0.86), B119 (0.87), B121 (0.88), S150 (0.0)], therefore being excluded for the endurance tests to bile salts, and ability to deconjugation.

**TABLE 1:** Survival rate, expressed as log cycle reduction, of strains isolated from açai fruits (*Euterpe oleracea*).

**Survival rate**	**Number of surviving isolates**	
	**pH 2.0 (*N* = 32)**	**Bile salts (0.3%) (*N* = 28)**
≥0.90	29 strains	26 strains
0.89–0.80	3 strains—B112 (0.86), B119 (0.87), B121 (0.88)	2 strains—A74 (0.89) and B139 (0.81)
≤0.79	1 strain—S150 (0.0)	–

### Resistance to Bile Salts

In the present study, 28 isolated strains that had a survival rate of more than 0.90 at pH 2.0 were tested for their ability to survive to the presence of bile salts (0.3%) during 4 h. The results (Supplementary Figure S2) showed that the viability of 7 strains (C21, C38, C39, B118, B134, B138, and Z183) remained stable (*p* > 0.05), while that of 11 strains was reduced (*p* < 0.05) (C37, C52, A73, A74, B122, B125, B137, B139, B140, B143, and Z190). However, most strains (26) had a survival rate greater than 0.90 (Table 1). Only two strains showed a lower rate [A74 (0.89) and B139 (0.81)], but still considered with a good viability (>50%).

### Ability to Deconjugate Bile Salts

The 28 strains assessed for their resistance to bile salts were tested for their ability to deconjugate bile salts on MRS agar plates containing 0.5% oxgall (data not shown). Thus, all strains evaluated in the present study had colonies with different morphologies compared to their corresponding controls. On agar containing 0.5% oxgall, the colonies were smaller and transparent, whereas they were white in the controls.

### Antimicrobial Activity Against Pathogens

Table 2 shows the results of the antimicrobial activity for 28 bacteria isolated from açai fruits against the pathogens *Escherichia coli*, *Salmonella* Typhimurium, *Enterococcus faecalis*, and *Staphylococcus aureus* using the spot-on-the-lawn method. The strains showed different levels of inhibitory action against the assayed pathogens. Among the 28 strains evaluated, 27 were able to inhibit the growth of all four pathogens. Strain B141 showed the greatest inhibitory capacity against *E. faecalis* compared to the other isolates and the reference probiotic. With respect to *S.* Typhimurium, 9 strains (C37, C39, A71, B109, B113, B125, B134, B135, and B140) showed a higher inhibitory capacity than the reference strain. Two strains (C21 and Z183) also exhibited a superior ability to inhibit *E. coli* compared to the reference strain, while 8 strains (A74, B125, B134, B135, B142, B143, Z188, and Z190) exhibited a superior ability to inhibit *S. aureus*. Among all tested isolates, strains B125, B134, and B135 showed the highest performance in inhibiting pathogens.

**TABLE 2:** Inhibition of pathogen growth by LAB strains isolated from açai fruits by agar test and identification of isolated lactic acid bacteria.

**Isolate**	Pathogenic species*				**Closest database match (accession number), identity (%)^#^**
	***EF***	***ST***	***EC***	***SA***	
C21	++	++	+++	++	*Pediococcus pentosaceus* (NR_042058.1), 100.0%
C52	++	++	−	++	
B109	++	+++	++	++	
B113	++	+++	++	++	
B125	++	+++	++	+++	
B134	++	+++	++	+++	
B137	++	++	++	++	
B138	++	++	++	++	
B139	++	++	++	++	
Z188	++	++	++	+++	
C39	++	+++	++	++	*Pediococcus pentosaceus* (NR_042058.1), 99.7%
A74	++	++	++	+++	
B122	++	++	++	++	
A71	++	+++	++	+	*Pediococcus pentosaceus* (NR_042058.1), 99.6%
B118	++	++	++	++	*Pediococcus pentosaceus* (NR_042058.1), 99.2%
C38	++	++	++	++	*Pediococcus pentosaceus* (NR_042058.1), 99.0%
A73	++	++	++	++	*Pediococcus pentosaceus* (NR_042058.1), 98.8%
B123	++	++	++	++	
C37	++	+++	++	++	*Pediococcus pentosaceus* (NR_042058.1), 98.5%
Z190	++	++	++	+++	*Pediococcus pentosaceus* (NR_042058.1), 98.3%
B135	++	+++	++	+++	*Lactiplantibacillus plantarum* (NR_104573.1), 100.0%
B140	++	+++	++	++	
B141	+++	++	++	++	
B142	++	++	++	+++	
B143	++	++	++	+++	*Lactiplantibacillus plantarum*(NR_115605.1), 100.0%
B144	++	++	++	++	
Z170	++	++	++	++	
Z183	++	++	+++	++	

*Pathogenic strains: EF, Enterococcus faecalis; ST, Salmonella Typhimurium; EC, Escherichia coli; SA, Staphylococcus aureus. ^#^Nucleotide sequences were submitted to National Center for Biotechnology Information and received corresponding GenBank accession numbers. The different scores reflect the different degrees of growth inhibition, measured in mm from the edge of the spot. (−) without inhibition. (+) zone of inhibition between 2 and 6 mm. (++) zone of inhibition between 7 and 11 mm. (+++) zone of inhibition between 12 and 16 mm.

### Identification of LAB Strains

Twenty-eight strains were identified as belonging to the genera *Lactiplantibacillus* and *Pediococcus* (Table 2). Sequencing of the 16S rRNA gene of these isolates showed that strains C21, C52, B109, B113, B125, B134, B137, B138, B139, and Z188 had 100% nucleotide homology with *P. pentosaceus*, while strains B135, B140, B141, B142, B143, B144, S150, Z170, and Z183 had 100% nucleotide homology with *L. plantarum*. In addition, strains C37, C38, C39, A71, A73, A74, B118, B122, B123, and Z190 had between 98.34 and 99.72% homology with *P. pentosaceus* (homology to GenBank sequences).

### Antagonistic Activity Against Pathogens in Açai Juice

**FIGURE 3:**
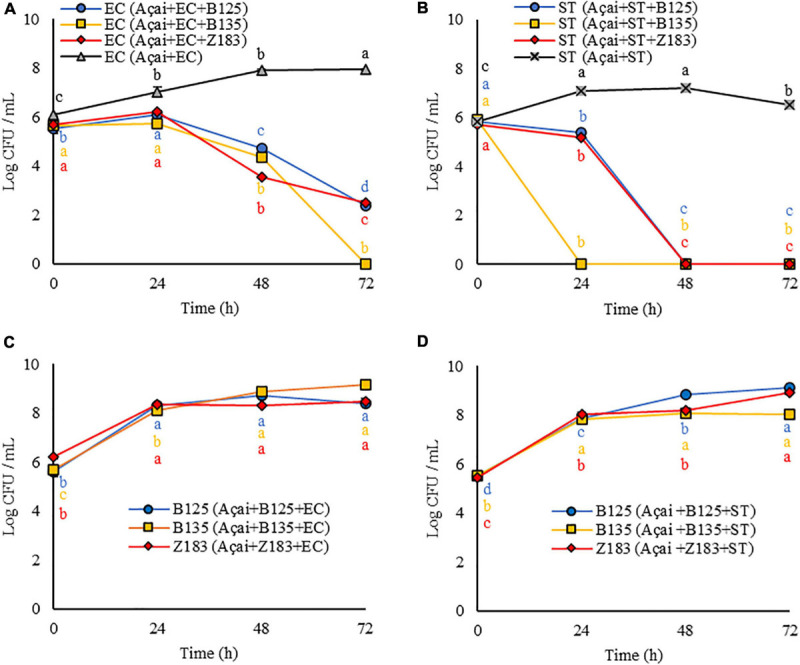
Response (log CFU/mL) of the antagonist activity of three lactic acid bacteria (B125, B135, and Z193) against pathogens **(A)** *Escherichia coli* (EC), and **(B)** *Salmonella* Typhimurium (ST), and LAB growth concomitant with the decrease in pathogen cell viability **(C)** EC, and **(D)** ST in açai juice during 3 days of storage at 25°C. Equal letters of the same color indicate no significant difference by Tukey test (*p* > 0.05).

To evaluate LAB strains’ antagonistic activity against pathogens, *P. pentosaceus* B125, *L. plantarum* B135, and *L. plantarum* Z183 isolates were selected. These three strains were attested as γ-hemolytic, coagulase negative, showed sensitivity or intermediate sensitivity for most antibiotics (AMP, GEN, TET, CLO, ERI, CLI, and RIF), except for the antibiotics VAN, CIP, and EST, which was expected. Survival rates were above 0.9 at pH 2.0 and 0.5% of bile salts and showed good antagonistic activity *in vitro* against pathogens. These strains were added in combination with the pathogens *E. coli* or *S.* Typhimurium in the açai juice, and the results are shown in Figure 3.

As shown in Figures 3A–D, the growth of both EC and ST was inhibited in the presence of the three LAB tested in the system (Açai + LAB + pathogen). Furthermore, LAB growth occurred at all evaluated times (24, 48, and 72 h) (Figures 3C,D) concomitant with the decrease in pathogen cell viability.

In the presence of *P. pentosaceus* B125 and *L. plantarum* Z183, the amount of *E. coli* was reduced from 5.5 to 2.4 log CFU/mL (*p* < 0.05) and from 5.7 to 2.5 log CFU/mL (*p* < 0.05) in 72 h (Figure 3A). The best result was obtained with *L. plantarum* B135, for which the initial quantity (0 h) of viable EC cells (5.7 log CFU/mL) was reduced below the limit of detection after 72 h (0.0 log CFU/mL) (*p* < 0.05) (Figure 3A).

The presence of LAB in açai was even more effective in inhibiting the growth of *S.* Typhimurium. When *P. pentosaceus* B125 and *L. plantarum* Z183 (Figure 3B) were tested, the initial quantities for both (5.8 log CFU/mL) were decreased below the detection limit by the plate count in 48 h (0.0 log CFU/mL) (*p* < 0.05). In the presence of *L. plantarum* B135, ST was no longer detected within 24 h by the plate counting method (*p* < 0.05) (Figure 3B).

It should be noted that both pathogens exhibited normal growth in açai juice, demonstrating, that there was an antagonistic activity of *P. pentosaceus* B125 or *L. plantarum* Z183.

### Preliminary Investigation of the Technological Properties of LAB With Probiotic Potential Based on pH for Addition to Food

**FIGURE 4:**
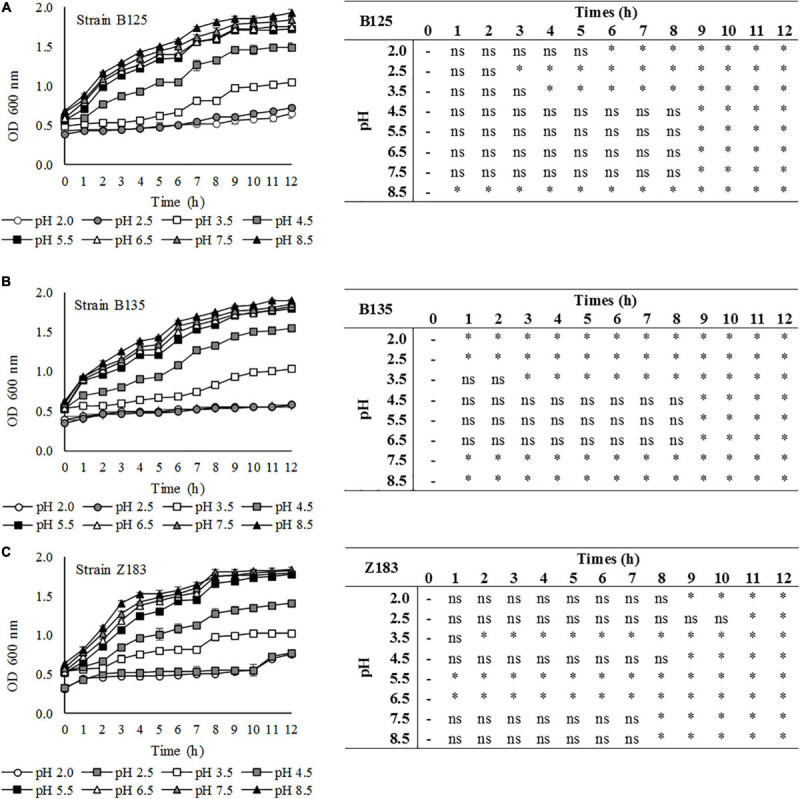
Tolerance of three lactic acid bacteria **(A)** B125, **(B)** B135, and **(C)** Z183 isolated from açai fruits (*Euterpe oleracea*) in MRS medium at different pHs (2.0–8.5). ^∗^Statistically significant variation in relation to time 0 at *p* < 0.05; ns, non-significant.

The growth of LAB that showed antagonistic activity against pathogens (*P. pentosaceus* B125, *L. plantarum* B135, and *L. plantarum* Z183) was evaluated in MRS medium with different pH values for 12 h through optical density analysis, and the results are shown in Figure 4. The results demonstrate that the three assayed LAB strains grew well on MRS broth with initial pH values of 4.5, 5.5, 6.5, 7.5, and 8.5. The growth of these bacteria was reduced at pH 3.5, and the strains showed lower tolerance at pH 2.5 and 2.0 but still showed growth, as revealed by the increase in optical density.

## Discussion

Research on microorganisms isolated from açai fruits remains scarce, with most studies focusing on the rapid decay of açai fruits with the quantification of molds, yeasts and fecal coliforms (Rogez et al., 2012). Aguiar et al. (2013) described an important property of lactic and acetic acid bacteria. Interestingly, a recent study focusing on the bacterial diversity of açai fruits identified more than 200 genera (Moura et al., 2018). Nevertheless, no isolation of the strains was performed, and only one strain of LAB has been identified to date (McCulloch et al., 2014).

Therefore, to the best of our knowledge, this is the first study on isolation and the genetic identification of a large number of endophytic LAB of açai fruits, the evaluation of probiotic characteristics, the safety aspects for their use, and their antagonist activity against pathogens associated with food-borne infections.

To guarantee the safety of the strains, even among a group of GRAS bacteria, it is necessary to assess the potential virulence of microorganisms (Joint FAO/WHO, 2002). Therefore, the eleven strains that gave a positive result for partial or total lysis of blood cells were eliminated from subsequent tests, as the absence of hemolytic activity is a selection criterion for potential candidate strains for application in food (Maragkoudakis et al., 2006).

Antibiotic resistance is one of the features that need to be analyzed because of concerns in the medical field regarding increasing levels of antibiotic resistance (Monteagudo-Mera et al., 2012). All tested strains (32) were resistant to vancomycin and streptomycin, as expected, as the literature reports that this characteristic is considered a natural or “intrinsic” property of LAB. Resistance to vancomycin has been reported to be due to the presence of D-alanine, with D-alanine ligase-related enzymes being used to separate them from other gram-positive bacteria (Klare et al., 2007), while intrinsic resistance to streptomycin (aminoglycoside) has been attributed to the absence of electron transport mediated by cytochrome, which lowers the permeability of cell membrane (Charteris et al., 2001; Danielsen and Wind, 2003).

The intrinsic resistance of LAB to ciprofloxacin (quinolone) still has an unknown resistance mechanism (Hummel et al., 2007). There are reports of a wide range of resistance to antibiotics in LAB, which reduces safety concerns (Ashraf and Shah, 2011), as according to Gueimonde et al. (2013), resistance to antibiotics becomes a safety concern when resistance is transferable, especially for pathogenic bacteria. In addition, this intrinsic resistance against antibiotics frequently used in human treatments, such as quinolones, glycopeptides and aminoglycosides, may be desirable since their beneficial use contributes to maintaining the balance of the gastrointestinal tract in cases of antibiotic-induced diarrhea (Charteris et al., 2001).

For the antibiotics CLO and ERI, all tested strains (32) showed sensitivity, while these strains exhibited sensitivity or intermediate sensitivity for PEN, AMP, GEN, TET, CLI, and RIF, with the exception of B137, which was resistant to TET. The resistance of the B137 strain to tetracycline must be further studied due to the risk of antibiotic resistance gene transfer since the genes for tetracycline resistance are plasmid encoded (Chuah et al., 2016). However, the detection of plasmid-transmitted antibiotic resistance genes, especially those for tetracycline, erythromycin, chloramphenicol and gentamicin has already been reported among LAB (Ouoba et al., 2008; Gueimonde et al., 2013). Given the antibiogram results observed in the present study, the risk of horizontal transfer of antibiotic-resistant genes to most identified LAB of açai fruits (31 strains) can be reduced, as they were susceptible to these antibiotics. To assess the probiotic potential of strains isolated from açai, the first test was to assess their tolerance to acidic conditions, as the pH of the stomach is generally from 2.5 to 3.5 and forms an effective barrier against the entry of external bacteria (Huang and Adams, 2004). Resistance to low pH is therefore considered an essential criterion for evaluating probiotic strains, since to reach the intestinal tract in adequate quantities, probiotics must first survive the acidic stomach conditions.

In the present study, among the 32 strains assessed in the low pH resistance test, 28 (87.5%) had a survival rate greater than 0.9 after 3 h at pH 2.0. Similar studies have shown that the viability of LAB strains between pH 2.0 and 4.0 represents an important performance criterion for a potential probiotic (Argyri et al., 2013). In addition, some studies have reported that survival rates above 0.9 at pH 2.0 constitute a good resistance to acids and represents an important criterion for LAB to have a beneficial effect on intestinal health (Liu et al., 2013; Almeida Júnior et al., 2015; Ferrari et al., 2016).

Variations in resistance to low pH have been observed among LAB. Presti et al. (2015) also evaluated the viability of LAB at pH 2.0 and observed that strains identified as *Lactobacillus acidophilus* PBS066 and *Lactobacillus plantarum* PBS067 had their viability decreased to undetectable levels just after 1 h and the *Limosilactobacillus fermentum* PBS073 strain after 2 h exposure. The strains *B. lactis* PBS075 and *B. longum* PBS108 were slightly affected, while *Limosilactobacillus reuteri* PBS072 and *Lacticaseibacillus rhamnosus* PBS070 showed the best survival rate during 3 h of exposure at pH 2.0.

The tolerance of strains to bile salts was tested, as these chemicals can damage the structure of the cell membrane and cause toxicity toward living cells (Yu et al., 2015; Han et al., 2017). Resistance to these salts is considered an important criterion for the selection of probiotic bacteria, as it allows them to survive, grow and function in the gastrointestinal tract (Oh and Jung, 2015; Yu et al., 2015; Plessas et al., 2017). The relevant physiological concentrations of human bile vary from 0.3 to 0.5% according to the time of digestion (García-Ruiz et al., 2014), where 0.3% (w/v) is an average concentration that has been generally established (Guo et al., 2012). Therefore, this concentration is used for the selection of resistant strains (Ramos et al., 2013).

The bile salt resistance test results showed that all strains remained viable under the tested conditions, with the lowest survival rate being 0.8 (B139). Mathara et al. (2008) observed good resistance of strains, which were tested in the presence of up to 0.3% bile salts and presented a growth rate above 0.5.

Our bile salt resistance test results of the present study were similar to those of Ramos et al. (2013), who demonstrated that all tested *Lactiplantibacillus* strains were able to grow in the presence of 0.3% (w/v) bile salts, although the time required to grow with and without bile was different between the isolates. They reported that strains identified as *L. plantarum* CH3, *L. plantarum* CH41 (isolated from cocoa fermentation) and *L. brevis* FFC199 (isolated from Cauim—an indigenous drink) had greater tolerance to bile salts, while other isolates identified as *L. fermentum* and *Levilactobacilus brevis* species had the lowest tolerance.

The strains isolated from açai fruits were exposed to bile salts for 4 h, which reflects the time required by food to reach the ileum. Interestingly, Oh and Jung (2015) showed that LAB can resist bile salts for longer times and that this resistance is different according to the tested strain.

Differences in tolerance, adaptation time and growth speed in medium with both low pH and bile salts are both species- and strain-specific (Maldonado et al., 2012; Montville and Matthews, 2013; Ramos et al., 2013).

Previous research has shown that the detoxification capacity of LAB cells occurs through the decay of bile salts, which is mediated by BSH enzymes produced by these strains (Bao et al., 2010; Yu et al., 2015). Indeed, some LAB strains secrete bile saline hydrolase, which hydrolyzes conjugated bile acids to release bile acids and amino acids (Begley et al., 2006; Sridevi et al., 2009; Franz et al., 2011).

Hamon et al. (2011) stated that tolerance of 3 *L. plantarum* strains to different concentrations of bile salts was correlated with the presence of six proteins (GshR1, GshR4, Cfa2, Bsh1, OpuA, and AtpH) that may be important for the response and adaptation to bile salts in *L. plantarum*. Therefore, the deconjugation of bile salts by enzymes produced by LAB strains can function as a detoxification mechanism that plays an important role in the tolerance to bile and the survival of the strain in the gastrointestinal tract (Begley et al., 2005; Ferrari et al., 2016).

The deconjugation of bile salts not only indicates the resistance and protection of LAB cells but has also been related to one of the beneficial effects attributed to probiotics, the reduction of serum cholesterol levels (Liong and Shah, 2005; Begley et al., 2006). As a result, strains isolated from açai fruits were also tested for their ability to deconjugate bile salts, and positive results were obtained for all 28 assayed strains.

Bile salt hydrolysis has been suggested to decrease serum cholesterol levels by increasing the demand for these compounds to synthesize new bile salts in the liver, increasing their excretion into stool as a result of decreased cholesterol solubility after deconjugation (Agaliya and Jeevaratnam, 2012; Lavilla-Lerma et al., 2013; Ferrari et al., 2016; Han et al., 2017).

The antimicrobial activity of the isolates obtained in the present study was investigated, and 27 of the 28 strains were able to inhibit the growth of the four pathogens to different degrees, where C52 was unable to inhibit *E. coli*.

Presti et al. (2015) previously reported on the capacity of LAB to inhibit pathogens, showing that *L. acidophilus* PBS066 and *L. plantarum* PBS067 strongly inhibited *E. faecalis*, *S. aureus*, *P. aeruginosa*, and *Escherichia coli*. The *L. rhamnosus* PBS070 and *Bifidobacterium* PBS075 and PBS108 strains inhibited *P. aeruginosa* and *E. coli*. *L. fermentum* PBS073 primarily had antimicrobial activity against *S. aureus*, *E. coli*, and *P. aeruginosa*, while *L. reuteri* PBS072 inhibited *S. aureus* and *P. aeruginosa*. Therefore, the authors suggested that the sensitivity of pathogens in antimicrobial activity tests depends on the type of LAB strains being evaluated, as was reported in other studies (Caggia et al., 2015; Yu et al., 2015).

Tejero-Sariñena et al. (2012) showed that strains that were putatively selected as probiotics inhibited the growth of *S.* Typhimurium, *E. coli*, *E. faecalis*, *S. aureus*, and *C. difficile*. The inhibitory effect that the authors observed this study was not attributed to the competition for substrates between probiotics and pathogens, as the bacteria had been previously killed with chloroform and due to the use of cell-free supernatants in a second agar diffusion method. When evaluating the pH of the supernatants, they observed that the lower the pH value, the greater the diameter of the zone of inhibition against the pathogenic indicator strains. Indeed, when investigating the organic acid profiles of glucose fermentation, they observed that lactic and acetic acids were produced in significant quantities, which may explain the inhibition zones, were the acidic environment was unsuitable for growth of pathogens.

The antimicrobial activity of LAB strains can be attributed to a number of compounds produced during their growth, including organic acids and bacteriocins (Zuo et al., 2016). Therefore, the ability to produce antimicrobial compounds can a key characteristic for the competitive exclusion of pathogens in the intestine and the effects of probiotic for the host (Salminen et al., 1998; Collado et al., 2005; Caggia et al., 2015).

The capacity of endophytic bacteria from açai fruits to inhibit pathogenic microorganisms should be emphasized. Indeed, açai juice could be associated with a high level of contamination. After collecting the fruits, their apex is severely damaged and comes into contact with different contaminated surfaces, which is responsible for presence of *E. coli* and sometimes *Salmonella* (Rogez et al., 2012). When these fruits are not correctly processed (i.e., washing and blanching at 80°C for 10 s or pasteurizing the açai juice), there is a high risk of food-borne infection among consumers, including by *Trypanosoma cruzi* (de Oliveira et al., 2019). In this context, noting that a portion of the Amazonian population consumes the sour açai drink (spontaneously fermented), the capacity of endophytic strains to inhibit pathogens in açai juice was verified. Interestingly, the selected LAB demonstrated ability to inhibit EC and ST in açai juice.

The antagonistic activity of LAB against pathogens in food was also assessed by Ferrari et al. (2016), who evaluated the inhibition of *Salmonella typhi* by autochthonous LAB isolated from goat dairy products and later added to artisanal goat cheese. Other studies have shown that indigenous microorganisms in fermented foods can improve the safety, technological and sensory properties, and shelf life of food products (Abriouel et al., 2008; Bao et al., 2012; Moraes et al., 2013).

Finally, a preliminary investigation of the technological properties of selected LAB was carried out at different pH values. For use as a probiotic, acid tolerance is essential to support the gastrointestinal environment, which is also an interesting feature for its use as dietary adjuvants in acidic foods (Minelli et al., 2004). Therefore, the growth of three LAB was tested for 12 h over a large range of pH values, from 2.0 to 8.5. The results were in agreement with those reported by Todorov et al. (2012), who used the same method and observed high levels of LAB growth at pH values of 5.0, 6.0, 7.0, and 9.0 and slight growth at lower pH values (4.0 and 3.0). Their results were also in agreement with those of Agaliya and Jeevaratnam (2012), showing a good growth of *Lactobacillaceae* strains in MRS broth with pH values of 3.5, 4.5, 7.5, and 8.5, while growth at pH 2.0 and 2.5 was affected.

Optical density-based analyses do not allow the viability of cells to be evaluated, only their density in the medium. However, due to the increase in OD, it is possible to confirm that the cells continued to multiply and were therefore viable.

## Conclusion

The *in vitro* characterization of endophytic LAB strains isolated from açai fruit in the present study showed several characteristics in common with probiotic bacteria. Indeed, most strains showed resistance to low pH and bile salts, which is predictive of their ability to resist passage through the gastrointestinal tract. The majority of strains showed a positive response regarding their safety through hemolytic activity and antibiotic susceptibility analyses, and the majority were also able to hydrolyze bile salts and inhibit the growth of pathogens, suggesting that they can survive and colonize the intestine and provide a benefit to the host.

The results of the present study showed that açai fruits are a potential source of LAB with interesting probiotic characteristics. The isolates *P. pentosaceus* B125, *L. plantarum* B135, and *L. plantarum* Z183 showed antibacterial activity against *E. coli* and *S.* Typhimurium *in vitro* and in açai juice. Thus, by carrying out complementary tests, these cultures could be used to inhibit the growth of pathogens in products to increase their functional value.

## Data Availability Statement

The datasets presented in this study can be found in online repositories. The names of the repository/repositories and accession number(s) can be found in the article/Supplementary Material.

## Author Contributions

SA and HR designed the study. SA and FG performed the collection and processing of the samples. SA and AL performed the experiments to evaluate the probiotic potential and antagonistic activity against pathogens of the isolated strains. SA and JMa performed the PCR analyzes. SA, RS, MN, and JMo performed sequencing analysis of the 16S gene. SA and JMa analyzed the diversity of the bacterial community. SA discussed the results. SA, JMa, FG, and HR revised the article. All authors contributed to the article and approved the submitted version.

## Conflict of Interest

The authors declare that the research was conducted in the absence of any commercial or financial relationships that could be construed as a potential conflict of interest.
