# Supplementary material for: Isolation and Genetic Identification of Endophytic Lactic Acid Bacteria From the Amazonian Açai Fruits: Probiotics Features of Selected Strains and Their Potential to Inhibit Pathogens
Source: Front Microbiol. 2021 Jan 8;11:610524. doi: 10.3389/fmicb.2020.610524 (PMC7819895; doi:10.3389/fmicb.2020.610524)
Supplement: Supplementary file 1 [file Data_Sheet_1.pdf]

Supplementary figure 1 - Survival ability of lactic acid bacteria isolated from açai (*Euterpe oleracea*) fruits at low pH (A, B, and C).

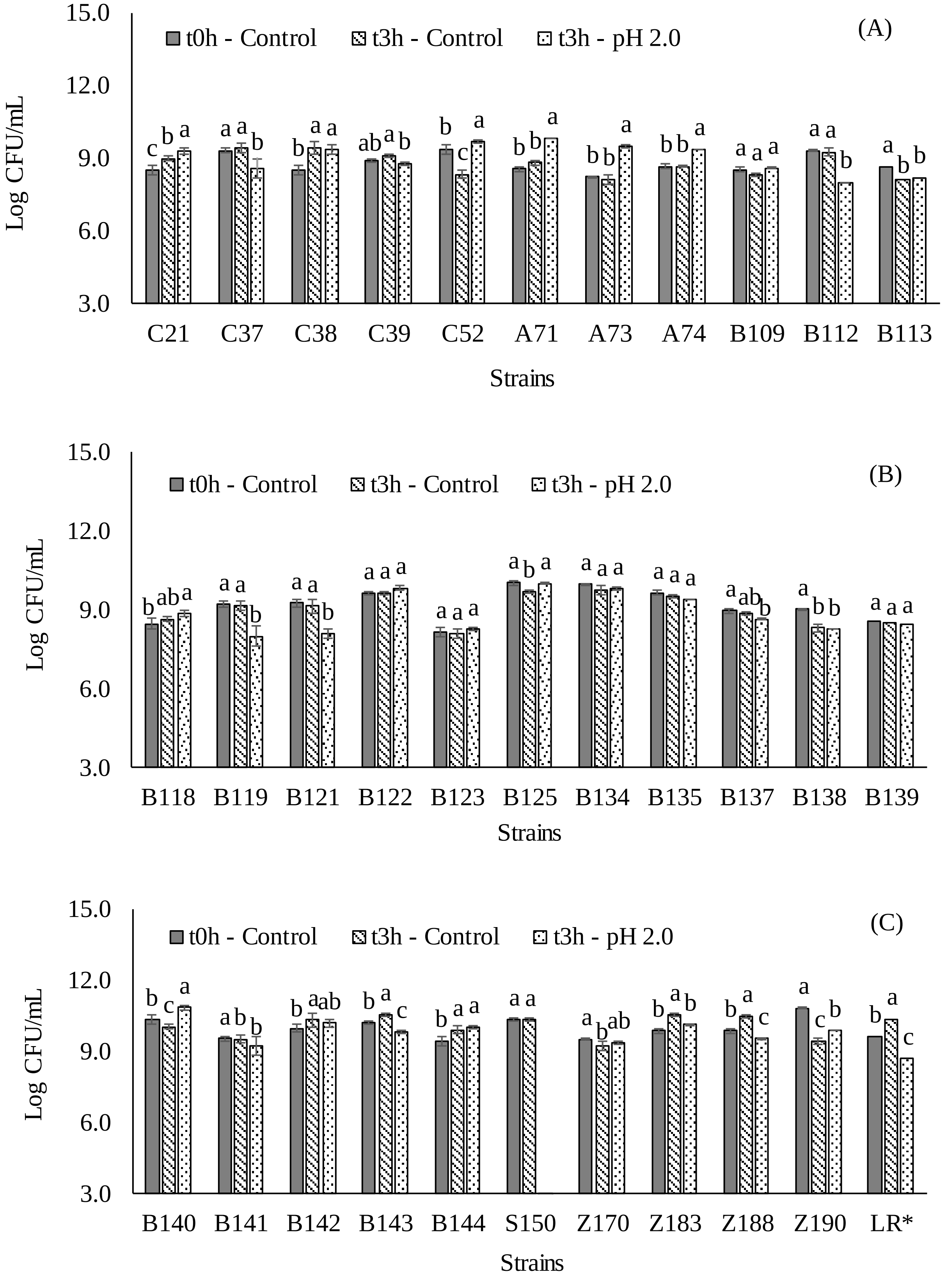

Triplicate test data, expressed as mean ± SD. Different lower case letters indicate significant ($p < 0.05$) difference. *LR - Reference strain - *Lacticaseibacillus rhamnosus*.
